# Interprofessional training for undergraduate rehabilitation therapy students at the University of the Witwatersrand enhancing cancer rehabilitation in South Africa

**DOI:** 10.4102/sajp.v82i2.2314

**Published:** 2026-04-30

**Authors:** Vaneshveri Naidoo, Kim Coutts, Merling Phaswana, Sonti Pilusa, Bhavna Bahgoo, Mary Lou Galantino, Nabeela Sujee

**Affiliations:** 1 Department of Physiotherapy, Faculty of Health Sciences, University of the Witwatersrand, Johannesburg, South Africa; 2 Department of Speech Pathology and Audiology, Faculty of Health Sciences, University of the Witwatersrand, Johannesburg, South Africa; 3 Department of Exercise Science and Sports Medicine, Faculty of Health Sciences, University of the Witwatersrand, Johannesburg, South Africa; 4 Department of Occupational Therapy, Faculty of Health Sciences, University of the Witwatersrand, Johannesburg, South Africa; 5 Physical Therapy Program, Stockton University, New Jersey, United States of America; 6 School of Medicine, University of Pennsylvania, Philadelphia, United States of America; 7 Centre for Health Science Education, Faculty of Health Sciences, University of the Witwatersrand, Johannesburg, South Africa

**Keywords:** undergraduate health professions education, interprofessional education, Health Science students, cancer rehabilitation, collaborative practice, healthcare team, patient-centred care

## Abstract

**Background:**

The national cancer strategy includes guidelines for rehabilitation as part of a collaborative team for cancer care. Interprofessional engagement is a critical attribute of a rehabilitation team. Thus, exposing students to live case scenarios in physiotherapy (PT), occupational therapy (OT) and speech-language pathology (SLP) provides development of Interprofessional Education Collaborative (IPEC) core competencies.

**Objectives:**

To evaluate students’ acquisition of interprofessional competencies (*Values and Ethics; Roles and Responsibilities; Interprofessional Communication; and Teams and Teamwork*) in cancer rehabilitation and explore their qualitative experiences of interprofessional engagement.

**Method:**

A cross-sectional survey design with open-ended questions was administered to final year rehabilitation students (PT, OT and SLP) post interprofessional education (IPE) session with a cancer survivor (CS).

**Results:**

Of 140 enrolled final year students, 64 completed the IPEC survey on Redcap. Descriptive and qualitative data analysis were conducted. High levels of agreement across all IPEC domains were observed, with the highest seen in *Teams and Teamwork*. Three themes, (1) Scope of practice, (2) Teamwork, (3) Experience of the IPE activity, emerged from the open-ended questions.

**Conclusion:**

High agreement across IPEC domains indicates enhanced collaborative attitudes and role clarity, despite limited exposure to oncology in participants’ undergraduate curricula. The presence of a CS added authenticity, fostering empathy and ethical practice. Students gained insight into professional boundaries, effective communication strategies and teamwork in complex care settings. Findings support integrating authentic, condition-specific IPE into curricula to strengthen collaboration and professional identity formation.

**Clinical implications:**

Undergraduate Health Sciences students can gain exposure to national cancer policies through IPE sessions. Future research can explore if the skills learnt translate to clinical teamwork post-graduation.

## Introduction

Cancer is a life-threatening and chronic condition that requires collaborative care (Department of Health 2015; Hudon et al. 2012). In South Africa, the National Cancer Strategic Framework for South Africa (2017–2022) underscores the importance of holistic cancer care from a interprofessional team (Department of Health 2015). Cancer and cancer treatment affect many bodily structures and systems resulting in long-term health complications such as pain, fatigue and limitations in functional ability (Silver 2017). Thus, undergraduate training must expose students to interdisciplinary and interprofessional collaboration across the cancer continuum.

Interprofessional education (IPE) has emerged as a crucial component in health professions education as a result of the increasing recognition of the complex nature of modern healthcare delivery (Ezzeddine & Price 2021; Mohammed, Anand & Saleena Ummer 2021; Olson & Bialocerkowski 2014). Interprofessional education aims to address this imperative by bringing together students from various healthcare professions to learn with, from and about each other, fostering mutual respect and understanding (Ezzeddine & Price 2021; Mohammed et al. 2021; Olson & Bialocerkowski 2014). Traditionally, healthcare professionals have operated within silos, with limited opportunities for collaboration and interdisciplinary understanding (Mohammed et al. 2021; Olson & Bialocerkowski 2014). However, the evolving healthcare landscape demands a paradigm shift towards teamwork, effective communication and shared decision-making among healthcare professionals (Mohammed et al. 2021; Olson & Bialocerkowski 2014). As healthcare becomes increasingly complex and interconnected, the integration of IPE into health professions education is essential for preparing future practitioners to meet the evolving needs of patients and communities effectively (Dyess et al. 2019).

Moreover, the evidence supporting the benefits of IPE continues to grow. There are numerous studies demonstrating the positive impact of IPE on student learning, patient outcomes and healthcare system efficiency (Mohammed et al. 2021; Olson & Bialocerkowski 2014). By exposing students to diverse perspectives and roles within the healthcare team, IPE enhances their ability to function effectively in interprofessional settings, ultimately improving patient safety and quality of care. Additionally, IPE promotes a culture of lifelong learning and collaboration among healthcare professionals, facilitating ongoing professional development and innovation (Mohammed et al. 2021; Olson & Bialocerkowski 2014; Van Diggele et al. 2020).

Although the benefits of IPE are well-documented (Patel et al. 2025), research exploring rehabilitation students’ perceptions of the influence of the IPE activity remains limited. Our study therefore aimed to investigate how key IPE competencies – Teams and Teamwork, Communication, Roles and Responsibilities and Values and Ethics – shape the learning experiences of rehabilitation students for the purpose of modelling best practice during clinical practice.

## Research methods and design

### Design

A cross-sectional survey design was conducted using a validated IPE instrument and student qualitative feedback.

### Setting

Our study was based at the University of the Witwatersrand (WITS) which is one of the seven universities in South Africa offering undergraduate training for physiotherapy (PT), occupational therapy (OT) and speech-language pathology (SLP). Approximately 194 rehabilitation therapists from PT, OT and SLP departments graduate each year at WITS.

### Population

We included final year undergraduate students from the three rehabilitation departments at WITS (PT, OT and SLP departments). There were 68 PT, 50 OT and 30 SLP students registered at the time of data collection (Table 1).

**TABLE 1:** Number of final year rehabilitation undergraduate students.

Rehabilitation profession	Number of students
Speech-language pathology	30
Occupational therapy	50
Physiotherapy	68
**Total**	**148**

### Procedure

#### Interprofessional education activity

Prior to the IPE event, an online information session was held, and all the facilitators from each rehabilitation profession were oriented to the objective of the IPE session and were trained with evidence-based facilitation techniques. Facilitators were reminded to foster a safe space for student engagement and interject with engaged dialogue when students asked for guidance.

The goal of the IPE session was to address all Interprofessional Education Collaborative (IPEC) competencies: Teams and Teamwork, Communication, Roles and Responsibilities and Values and Ethics. Students received a brief introduction prior to meeting the cancer survivor (CS), which included the session’s objectives, a video link on IPE and profession-specific instructions via an activity map. Investigators informed students about the Interprofessional Education Collaborative (IPEC) competency survey which was completed voluntarily after the session.

Final year students from PT, OT and SLP gathered in a central venue at a higher education institute in South Africa. The students had name tags and stickers to identify the various professions; the PT students (blue), OT (green) and SLP students had pink stickers. A CS shared her experience of the diagnosis, gaps and health challenges related to cancer and its treatment. Through a biopsychosocial approach, the CS provided a detailed history of her condition and the impact it had on her life. She succinctly described the healthcare services she received as well as the care and support she wished to have experienced. Students had an hour to interact with a CS. Students had the opportunity to ask questions. They were allowed to ask the CS questions where they needed clarity to further understand pathology, surgical procedures and follow-up treatment interventions to address her cancer trajectory.

The students were equally divided into teams with diverse professions, 10 groups of 13–15 students. The interprofessional groups (PT, OT, SLP) met at their specific venue and were allocated a facilitator to moderate and guide the discussion.

The group session included several interactive activities designed to enhance collaborative learning. Students began by mapping the care activity and uploading their reflections on Flipgrid, an online platform for sharing short videos. The students engaged in a discussion about the specific roles of each rehabilitation professional in patient care, identifying key assessments and brainstorming a comprehensive rehabilitation intervention plan that addressed both patient and family needs, while considering all relevant influencing factors. Finally, the session concluded with a debrief, allowing students to reflect on the IPE experience and the lessons learned.

#### Data collection and instruments

After the session, the students were given a link to the validated IPEC survey (original version) to complete. The original IPEC contains 42 items and assesses interprofessional collaboration competences, focusing on four domains: Values and Ethics for Interprofessional Practice (10 items); Roles and Responsibilities (nine items); Interprofessional Communication (11 items); and Teams and Teamwork (12 items). Each item uses a 5-point agreement scale ranging from strongly disagree ‘1’ to strongly agree ‘5’. Mean or median scores are calculated for each domain. Higher scores indicate greater self-perceived competence in that domain (IPEC Competency Self-Assessment Tool; National Center for Interprofessional Practice and Education 2016).

Based on the indicators calculated to perform a psychometric investigation of the IPEC survey, it has acceptable reliability and validity with Cronbach’s Alpha determined at 0.84 (Dow et al. 2014; Lockeman et al. 2016; Rasouli et al. 2024). The tool evaluated interprofessional collaboration competency between medicine and nursing across the four areas and can evaluate interprofessional competency as a valid and reliable questionnaire. Interprofessional education collaborative can be used to plan scaffolding of learning across healthcare professional training (Rasouli et al. 2024).

Open-ended questions on the most valuable or useful aspect of the IPE experience and future suggestions to improve the IPE experience were added to the IPEC survey.

### Data analysis

All data were captured using the online password-protected Research Electronic Capture System (REDCap; Vanderbilt University, Nashville, US), which was only accessible to the research team and analysed descriptively (frequency and percentage), and median and interquartile range (IQR) for each domain as data was not normally distributed, using Statistica version 14.2 (StataSoft Inc., Tulsa, OK, United States [US]). The categories of strongly agree and strongly disagree were integrated with agree and disagree, respectively.

Students’ responses were analysed via inductive thematic analysis. Two authors (Vaneshveri Naidoo and Merling Phaswana) read all the responses independently and generated themes from the data; these were further discussed until a consensus was reached. Microsoft Excel (version 11; Microsoft Corp., Redmond, WA, US) spreadsheet was used to manage and analyse the data. A high level of agreement is indicated when 70% or more of students concur with the statement. A moderate level of agreement is defined as 50% – 69% concurrence, while a low level of agreement is characterised by less than 50% concurrence with the statement.

### Ethical considerations

Ethical clearance to conduct our study was obtained from the WITS Human Research Ethics Committee (Non-Medical) (No. H24/07/30). Student information was kept confidential and completion of the survey was anonymous. All participants provided informed consent.

## Results

A total of 140 final year Health Sciences students from three rehabilitation professions participated in the 2024 IPE activity, which included 68 PT, 50 OT and 30 SLP students, respectively.

### Interprofessional education collaborative competency

The results are presented across the four IPEC domains: *Values and Ethics; Roles and Responsibilities; Interprofessional Communication and Teams and Teamwork*. Most students agreed across all four domains of the IPEC survey.

### Values and ethics domain

Table 2 presents the median score for this domain 49.0 (IQR: 43.0–50.0), indicating a high level of agreement with ethical interprofessional practice. Most participants agreed with all items, with agreement ranging from 90.1% to 96.8% on competencies such as patients at the centre of interprofessional healthcare delivery, diversity appreciation, ethical conduct and competence. A minority ≤ 6.3% neither agreed nor disagreed with the statements.

**TABLE 2:** Students’ values and ethics in interprofessional education.

Values and ethics domain	Disagree		Neither agree nor disagree		Agree	
	*n*	%	*n*	%	*n*	%
1. Place the interests of patients at the centre of interprofessional healthcare delivery.	3	4.8	0	-	60	95.2
2. Respect the privacy of patients while maintaining confidentiality in the delivery of team-based care.	3	4.8	3	4.8	57	90.1
3. Embrace the diversity that characterises patients and the healthcare team.	1	1.6	1	1.6	61	96.8
4. Respect the unique cultures, values, roles/responsibilities and expertise of other health professions.	1	1.6	2	3.2	60	95.2
5. Work in cooperation with those who receive care and those who provide support or care.	1	1.6	4	6.3	58	92.1
6. Develop a trusting relationship with patients, families and other team members.	1	1.6	4	6.3	58	92.1
7. Demonstrate high standards of ethical conduct and quality of care in my contributions to team-based care.	1	1.6	3	4.8	59	93.6
8. Manage ethical dilemmas specific to interprofessional patient-centred care situations.	1	1.6	4	6.3	58	92.1
9. Act with honesty and integrity in relationships with patients, families and other team members.	0	-	2	3.2	61	96.8
10. Maintain competence in my own profession appropriate to my scope of practice or level or training.	0	-	2	3.2	61	96.8

Note: Median (IQR): 49.0 (43.0–50.0).

IQR, interquartile range.

### Roles and responsibilities domain

Table 3 presents the median score for this domain: 42.5 (IQR: 37.7–45.0). Agreement levels remained high, with most items receiving over 90% agreement. The highest agreement (96.8%) was in communication with team members to clarify responsibilities in executing treatment plans or interventions. Recognising limitations, engaging diverse professionals and establishing interprofessional relationships as well as engaging in continuous professional development were also emphasised.

**TABLE 3:** Students’ roles and responsibilities in interprofessional education.

Roles and responsibilities domain	Disagree		Neither agree nor disagree		Agree	
	*n*	%	*n*	%	*n*	%
11. Communicate my roles and responsibilities clearly to patients, families and other professionals.	3	4.8	1	1.6	59	93.6
12. Recognise my limitations in skills, knowledge and abilities.	1	1.6	2	3.2	60	95.2
13. Engage diverse healthcare professionals with complementary professional expertise to develop strategies to meet specific patient care needs.	0	-	4	6.3	59	93.6
14. Explain the roles and responsibilities of other care providers and how the teamwork together to provide care.	3	4.8	3	4.8	57	90.1
15. Use the full scope of knowledge, skills and abilities of available health professionals and healthcare workers to provide care that is safe, timely, efficient, effective and equitable.	1	1.6	3	4.8	59	93.6
16. Communicate with team members to clarify each member’s responsibility in executing components of a treatment plan or public health intervention.	0	-	2	3.2	61	96.8
17. Establish interprofessional relationships to improve care and advance learning.	2	3.2	1	1.6	60	95.2
18. Engage in continuous professional and interprofessional development to enhance team performance.	0	-	3	4.8	59	95.2
19. Use unique and complementary abilities of all members of the team to optimise patient care.	1	1.6	3	4.8	58	93.6

Note: Median (IQR): 42.5 (37.7–45.0).

IQR, interquartile range.

### Interprofessional communication domain

This domain recorded the second highest median score of 52.0, with an IQR of 45.0 to 54.3, as shown in Table 4. High levels of agreement were observed in effective communication, active listening and conflict resolution. Communicating with respect and valuing collaboration was scored 96.8%. Interestingly ‘Avoid discipline-specific terminology when possible’ and ‘Give timely, sensitive feedback to others about their performance on the team’ were scored 82.5 and 84.1, respectively.

**TABLE 4:** Students’ interprofessional communication in interprofessional education.

Interprofessional communication domain	Disagree		Neither agree nor disagree		Agree	
	*n*	%	*n*	%	*n*	%
20. Choose effective communication tools and techniques to facilitate discussions and interactions that enhance team function.	2	3.2	1	1.6	60	95.2
21. Communicate information with patients, families and healthcare teams members in a form that is understandable.	2	3.2	2	3.2	59	93.6
22. Avoid discipline-specific terminology when possible.	1	1.6	10	-	52	82.5
23. Express my knowledge and opinions to team members involved in patient care with clarity and respect.	2	3.2	0	-	60	96.8
24. Listen actively and encourage ideas and opinions of other team members.	0	-	3	4.8	60	95.2
25. Give timely, sensitive feedback to others about their performance on the team.	4	6.3	6	9.5	53	84.1
26. Respond respectfully to feedback from others on my healthcare team.	1	1.6	2	3.2	60	95.2
27. Use appropriate, respectful language in a given difficult situation such as interprofessional conflict.	0	-	5	7.9	58	92.1
28. Recognise how my experience and expertise contribute to communication, conflict resolution and interprofessional working relationships.	0	-	4	6.3	59	93.6
29. Recognise how my position in the hierarchy of the healthcare team, contributes to communication, conflict resolution and interprofessional working relationships.	0	-	4	6.3	59	93.6
30. Consistently communicate the importance of teamwork in patient-centred and community-focused care.	0	-	2	3.2	60	96.8

Note: Median (IQR): 52.0 (45.0–54.3).

IQR, interquartile range.

### Teams and teamwork domain

The highest median score was observed in this domain (Median: 55.0, IQR: 48.0–60.0), with an 87.1% – 96.8% level of agreement as seen in Table 5. High agreement levels, above 90%, were seen in teamwork strategies, shared accountability and reflection on performance. Interestingly, the highest score (96.8%) was achieved in: *perform effectively on teams and in different team roles in a variety of settings*. Though minimal, disagreement or neutrality were also noted within this domain. Disagreement or neutrality, though negligible, was observed in ‘Reflect on my healthcare team’s performance for my team’s improvement’, ‘Apply leadership practices that support collaborative practice and team effectiveness’ and ‘Engage others to constructively manage disagreements that arise between healthcare professionals, patients, and families’.

**TABLE 5:** Students’ teams and teamwork in interprofessional education.

Teams and teamwork domain	Disagree		Neither agree nor disagree		Agree	
	*n*	%	*n*	%	*n*	%
31. Describe the process of team development.	2	3.2	6	9.7	54	87.1
32. Describe the roles and practices of effective healthcare teams.	1	1.6	4	6.3	57	91.9
33. Engage other health professionals in shared problem solving appropriate to the specific care situation.	2	3.2	3	4.8	57	91.9
34. Inform care decisions by integrating the knowledge and experience of other professions appropriate to clinical situation.	1	1.6	2	3.2	59	95.2
35. Apply leadership practices that support collaborative practice and team effectiveness.	1	1.6	6	9.5	56	88.9
36. Engage others to constructively manage disagreements that arise between healthcare professionals, patients, and families.	3	4.8	4	6.3	56	88.9
37. Share accountability with other professions, patients, and communities for outcomes relevant to prevention and health care.	2	3.2	3	4.8	58	92.1
38. Reflect on my individual performance for my improvement.	2	3.2	2	3.2	59	93.6
39. Reflect on my healthcare team’s performance for my team’s improvement.	4	6.3	3	4.8	56	88.9
40. Use strategies that will improve the effectiveness of interprofessional teamwork and team-based care.	2	3.2	2	3.2	59	93.6
41. Use available evidence to inform effective teamwork and team-based practices.	2	3.2	4	6.3	57	90.1
42. Perform effectively on teams and in different team roles in a variety of settings.	1	1.6	1	1.6	60	96.8

Note: Median (IQR): 55.0 (48.0–60.0).

IQR, interquartile range.

### Students’ reflections on the novel interprofessional education activity

The students were asked to reflect on their IPE experience. We identified three key themes from participants’ open-ended responses, based on how often they were mentioned and what they revealed about people’s experiences. These themes were (1) experience of the IPE activity; (2) scope of practice; and (3) teamwork.

#### Theme 1: Experience of the interprofessional education activity

Students enjoyed the IPE activity and found it very insightful:

‘[*A*] very helpful and exciting way of learning and creating knowledge.’ (Participant 68)

‘Really enjoyed the session, it was interesting to work with the other professions.’ (Participant 56)

#### Theme 2: Scope of practice

Students felt that they learnt about each other’s role and responsibilities in patient care:

‘I now know and understand the roles of the other professionals in the rehab programme.’ (Participant 65)

Students emphasised the importance of referral and optimal patient care:

‘It helps a lot in knowing the different roles of other health care professionals, thus making the referral system easier and knowing how to work together for the better of the patient.’ (Participant 59)

‘It was great to engage with PT, OT, and ST all together, and to identify how we can all help build on each other’s skills/scope to provide the best care for our patients. I feel that I now have a better understanding on when and how I can work together with a PT or ST [*I am an OT*], or what aspects I can refer to them for to give a patient the best chance of a good functional outcome.’ (Participant 63)

Students also expressed that this insight challenged their preconceived notions and helped them let go of certain myths and biases:

‘I was also able to better understand what the other professions do, and we were able to debunk some myths and overcome biases. It was a very insightful session.’ (Participant 63)

#### Theme 3: Teamwork

Students highlighted the value of effective teamwork and advocacy and how these aspects enhanced their mutual understanding:

‘The teamwork was excellent.’ (Participant 55)

‘I enjoyed seeing how our professions interlink and how we can advocate for each other more.’ (Participant 72)

Students appreciated the opportunity to collaborate with professionals from other rehabilitation professions:

‘Really enjoyed working on a case alongside the other two professionals present in our discussion today. It was interesting to see how we each sort complement each other yet in practice we sometimes struggle to see that clearly outlined. But I enjoyed today’s discussion with the other team members.’ (Participant 58)

The student’s reflection derived from the themes illustrates the core IPE values of Roles and Responsibilities, Teams and Teamwork and Communication seen in Table 2 to Table 5.

## Discussion

This study examined interprofessional collaborative competencies among rehabilitation students including PT, OT and SLP, following participation in a cancer case-based IPE learning activity. Overall, high levels of agreement across all four IPEC competency domains, *Values and Ethics, Roles and Responsibilities, Interprofessional Communication and Teams and Teamwork*, suggest that the case scenario was effective in promoting attitudes consistent with collaborative, patient-centred practice. Furthermore, students reported competence in discussing their roles as well as other team members’ roles within cancer rehabilitation, despite cancer care not being embedded in their undergraduate training. The IPE learning activity also displayed contextual relevance to the South African context, by making use of an in-person CS as part of the IPE experience.

### Values and ethics

Cancer care requires high levels of sensitivity and empathy with patients and family members, from healthcare professionals. Maintaining a patient-centred approach can be difficult when a patient’s choices or preferences differ from the healthcare professional’s own values or beliefs (Moore et al. 2025). The IPE activity results with a median score of 49.0 (IQR: 43.0–50.0) and agreement rates exceeding 90% indicate a strong collective orientation towards ethical interprofessional practice. This aligns with prior IPE research showing that values such as patient-first decision-making, professional integrity and commitment to competence form the foundation of sustainable collaborative work, especially in complex, high-stakes care such as oncology (Chamala et al. 2021; Sands, Stanley & Charon 2008; Uslu-Sahan & Terzioglu 2020). In the South African context, where interprofessional cancer care may be constrained by uneven resource distribution, these attitudinal strengths are critical for driving equity and patient advocacy. Providing an opportunity for exposure to various members of the team in an oncology case empowers students to ensure holistic care for their patients (Rathore et al. 2025).

### Roles and responsibilities

Rehabilitation is an important phase in the cancer care continuum, having a interprofessional approach throughout survivorship is important (Rathore et al. 2025). The IPE activity provided an opportunity for students to explore and advocate for their own role in the care of the patient as well as learn about the roles of other rehabilitation professions. It also highlighted grey areas where there is an overlap in scope, and students had the time to practice how to navigate this within the client’s best interest. High agreement across domains suggests that the cancer case successfully illuminated the distinct yet complementary contributions of each profession. This role of clarity is particularly important in oncology, where care is often prolonged and emotionally demanding, requiring seamless role transitions and mutual respect. For rehabilitation students, such clarity during training can pre-empt the professional silos that sometimes persist in practice (Soubra et al. 2018). The opportunity to navigate grey areas and advocate for their own roles during the IPE activity also equipped students with skills to navigate the common silo-approach as clinicians, and ensure a richer, more efficient approach to patient care.

### Communication and team function

The emphasis placed on interprofessional communication and teamwork is especially relevant in cancer care, where treatment plans are iterative and often involve complex decision-making (Fragner et al. 2024). In our study, the cancer case provided a shared clinical anchor that appeared to enhance students’ appreciation for open information exchange, conflict resolution strategies and co-creation of care plans. These new skills aligned with the benefits described in global IPE literature, but assumed additional significance given South Africa’s diverse linguistic and cultural landscape, where cultural competence is a crucial complement to clinical expertise (Matthews & Van Wyk 2018). During the IPE activity, students became increasingly aware of how their profession-specific jargon could be an obstacle in interprofessional team discussions. Explaining concepts unique to their profession was a skill that may not necessarily have been highlighted outside of this IPE activity.

### Educational significance

Embedding IPE within a cancer care context may have amplified the learning impact by situating collaboration in a high-empathy, high-complexity clinical frame. Cancer, as a ‘whole person’ condition with significant psychosocial dimensions, naturally necessitates coordinated rehabilitation health professional input (Sulosaari et al. 2024). The observed attitudinal gains across all IPEC domains support the integration of authentic, condition-specific cases into undergraduate curricula, not as isolated events, but as longitudinal threads that reinforce collaborative practice patterns (IPEC 2023b). This could also potentially assist with professional identity training and formation but needs to be explored. Considering the pressure to cover many complex diagnoses within the various undergraduate programmes, the cancer-specific IPE activity was a valuable exercise in increasing knowledge on cancer rehabilitation and improving awareness of the various rehabilitation professions and their roles in cancer care.

### Limitations and future direction

While the strong agreement rates are promising, our study only assessed self-reported perceptions and was limited in including observed collaborative behaviour. Therefore, the data could be skewed by perceptions related to the use of IPE rather than determine students’ ability to work collaboratively (Yates, Gough & Brazil 2022). However, the use of the IPEC competency self-assessment tool highlighted and confirmed student experiences in the four IPEC domains.

Future research could employ mixed methods by integrating objective team performance measures, reflective narratives or simulated patient feedback, to more robustly capture competency development. The professional degree programmes included departments from the Faculty of Health Sciences and Humanities. The scope was limited because other professions, including medical students, were unavailable at the time of our study. Expanding the scope to include nursing, dietetics and medical students may also enrich role understanding and further reflect real-world oncology teams. There is also a need for longitudinal studies. Considering the high level of agreement observed, it would be valuable to explore whether these IPE competencies are sustained when students are working in their respective healthcare setting. Further translational research is needed.

## Conclusion

Our study demonstrated that a cancer case-based IPE activity involving PT, OT and SLP students in South Africa was associated with high self-reported competency across all IPEC domains. Emphasising values, ethics, roles, communication and teamwork, the IPE activity highlighted the importance of fostering collaborative attitudes during undergraduate training. For example, in oncology where patient needs are multifaceted, prolonged and resource-constrained, teamwork competencies are essential for effective, patient-centred care. Embedding IPE learning within a real-world cancer case reinforced the relevance of each profession’s contribution, strengthened problem-solving skills, and enhanced clinical reasoning competencies for future cancer rehabilitation practice. While these findings are promising, further research is needed to explore how such skills translate into clinical settings, sustain into early career practice and expand to include additional healthcare professions, thereby enriching the experience and better reflecting the realities of cancer care in South Africa.
